# Transcriptome analyses of urine RNA reveal tumor markers for human bladder cancer: validated amplicons for RT-qPCR-based detection

**DOI:** 10.18632/oncotarget.27954

**Published:** 2021-05-11

**Authors:** Josephine Dubois, Jacqueline Rueger, Bernhard Haubold, Rosel Kretschmer-Kazemi Far, Georg Sczakiel

**Affiliations:** ^1^Institut für Molekulare Medizin, Universität zu Lübeck and UKSH, Campus Lübeck, Lübeck D-23538, Germany; ^2^Max-Planck-Institute for Evolutionary Biology, Department of Evolutionary Genetics, Ploen 24306, Germany

**Keywords:** tumor diagnostics, non-invasive, urine RNA, biomarker, transcriptome analysis

## Abstract

Non-invasive clinical diagnostics of bladder cancer is feasible via a set of chemically distinct molecules including macromolecular tumor markers such as polypeptides and nucleic acids. In terms of tumor-related aberrant gene expression, RNA transcripts are the primary indicator of tumor-specific gene expression as for polypeptides and their metabolic products occur subsequently. Thus, in case of bladder cancer, urine RNA represents an early potentially useful diagnostic marker.

Here we describe a systematic deep transcriptome analysis of representative pools of urine RNA collected from healthy donors versus bladder cancer patients according to established SOPs. This analysis revealed RNA marker candidates reflecting coding sequences, non-coding sequences, and circular RNAs. Next, we designed and validated PCR amplicons for a set of novel marker candidates and tested them in human bladder cancer cell lines. We identified linear and circular transcripts of the S100 Calcium Binding Protein 6 (S100A6) and translocation associated membrane protein 1 (TRAM1) as highly promising potential tumor markers.

This work strongly suggests exploiting urine RNAs as diagnostic markers of bladder cancer and it suggests specific novel markers. Further, this study describes an entry into the tumor-biology of bladder cancer and the development of gene-targeted therapeutic drugs.

## INTRODUCTION

Human bladder cancer (BCa) is one of the most common cancer types in humans. It is the 6th most common malignancy in men and the 10th most common tumor type worldwide [1, 2]. In 2020, BCa accounted for 4.4% of all male tumors worldwide [2]. BCa is the cause of a high number of mortalities [3] and BCa is related to high health care costs [4]. While there is extensive biomedical research on diagnostic and therapeutic tools for BCa, incidence remains high. Hence, improved diagnostic approaches are required for diagnosing new cases and for therapy monitoring.

For human BCa, the diagnostic gold standard is based on cystoscopy. However, this is invasive, bears a certain health risk, and is related to relatively high costs [5, 6]. Alternatively, cytological evaluation is non-invasive and provides specific information but it seems to fail to detect low-grade BCa sensitively enough [7]. With regard to BCa, RNA markers have been studied by transcriptome analyses of single cells [8], tissue samples [9] and urine [10]. While it does not seem to be realistic that cystoscopy will be fully replaced in the near future, additional independent diagnostic parameters are likely to substantially improve detection of BCa and therapy monitoring [11, 12].

Thus, there is a need for alternative and powerful diagnostic tools. Ideally, these should provide non-invasive access to tumor markers, increased sensitivity and specificity, and primary human samples should be easily available. These requirements are met by most of liquid biopsies, i.e., body-fluids including blood, plasma, serum, urine, and sputum. In case of BCa, urine is thought to be the most promising source for RNA-based tumor markers as direct physical contact between tumor cells and urine seems to be given. Urine as a source of markers is particularly attractive because urine is easily available and the detection of molecular markers is already established or seems to be feasible [13, 14]. This includes systematic and frequent screens of potential patients of BCa or a follow-up of patients after therapy.

While urine contains detectable macromolecular markers, the concentrations are very low when compared to tissue samples or circulating cells in blood [15, 16]. This is particularly true for nucleic acids, i.e., for DNA and RNA [16]. In case of RNA, one usually finds concentrations of total RNA in the nanogram or even the sub-nanogram range per 10 mL of urine [15]. This is supported by indirect evidence suggesting amounts of urine RNA that can only be detected by RT-qPCR [17, 18]. Usually, these concentrations are too low for detection by standard analytical methods and even after preparation, RNA solutions are extremely diluted. Further, the inter-donor variability of urine RNA concentrations can be extremely high ranging over up to four orders of magnitude.

On the other hand, urine RNA samples contain RNAs which are suitable for cDNA synthesis and RT-qPCR detection [16]. Cell-free RNA contained in urine samples can be protected against degradation, i.e., stabilization of RNA markers is possible such that shipping of samples and storage is possible. In technical terms, it might be an advantage to avoid negative effects mediated by large amounts of proteins and lipoproteins on other kinds of biopsies which could interfere during RNA preparation and subsequent steps.

The proof-of-concept of a non-invasive and RNA-based diagnostic of BCa has been described by a number of studies [19, 20]. However, most of them were based on intuitive marker selection [21], or on large but incomplete or biased sets of marker candidates [19, 22]. Initial studies indicated promising RNA marker candidates and provided functional biological insights and interactions of tumor markers [10, 23, 24].

More recent studies describe a search for RNA-based tumor markers by network analyses which indicate marker candidates [22] and by attempting to include the complete RNA sequence content in an unbiased fashion. As a result, larger sets of long-chain non-coding RNA sequences and circular RNA (circRNA) were identified and suggested as marker candidates for BCa [25, 26]. CircRNAs are promising biomarkers in liquid biopsies, as they are highly resistant to RNase activity because the lack of free ends [27, 28]. Furthermore, they often show tissue- and development stage-specific expression [29]. This reflects the increasing role of circRNAs for malignant cell proliferation and, hence, as diagnostic tools [30, 31].

In this study we describe a systematic, whole transcriptome analysis of urine RNA aiming to identify differentially expressed transcripts between healthy individuals and high risk (HR) BCa patients. This consists of SOP-based acquisition of urine, stabilization of urine RNA, delivery and storage of samples, RNA preparation, and deep transcriptome analyses.

## RESULTS

### Concept underlying this study

The possibility to stabilize urine samples, to ship them to diagnostic laboratories without degradation, and to analyze the composition of cell-free and cell-associated RNA enables one to search for RNA-based markers of tumors, primarily tumors of the urinary tract. In order to bypass the technical problem of very low RNA concentration in urine RNA samples which is not sufficient for standard transcriptome analyses, we decided to pool seven samples of urine RNA (Supplementary Table 1). These samples represent the mean values of a set of markers determined recently in a large set of urine samples, i.e., 47 patients within a control group (C) and 66 samples of the high risk (HR) patient group. Markers include 18S rRNA, Keratin 20 (KRT20), LIM and SH3 protein (LASP1), Oncoprotein 18 (OP18), Uroplakin 1A (UPK1A), and Baculoviral IAP repeat-containing protein 5 (BIRC5) (Table 1).

**Table 1:** Composition of pools C and HR

Pool	Patient #	KRT20	LASP1	OP18	UPK1A	BIRC5	18S rRNA	Mass in pool [ng]	Proportion in pool [%]	Final pool concentration
**C**	48	1	1,751	336	178	56	3,320,989	~16	15.24	1 ng/μl
	50	0	91	16	112	3	510,671	~18	17.14	
	80	32	1,728	23	456	7	1,009,850	~16	15.24	
	138	92	905	3	628	2	409,198	~13	12.38	
	160	3,911	3,081	2,141	5,109	151	1,184,859	~16	15.24	
	166	8	363	27	204	1	193,374	~15	14.29	
	170	10	1,338	36	79	2	607,790	~11	10.48	
**HR**	41	7,477	4,758	2,080	28,110	446	12,610,137	~18	15	2.6 ng/μl
	46	752	606	1,850	13,291	208	1,803,240	~16	13.33	
	107	91	3,361	10	110	0	1,452,569	~18	15	
	128	9,966	2,388	777	5,632	166	2,329,364	~19	15.83	
	159	1,640	3,350	325	5,385	67	1,585,600	~16	13.33	
	164	3,492	2,507	411	3,193	33	1,538,373	~17	14.17	
	183	42	5,725	2,756	1,907	346	2,816,141	~16	13.33	

All samples in pools C and HR were collected from control individuals and patients with high risk bladder cancer, respectively. Shown are the RNA expression levels of five potential biomarkers and 18S rRNA as determined by RT-qPCR. Mass and percentage contribution of each individual sample within the final pool is shown in addition to the final pool concentrations in ng/μl.

Next, urinary RNA was isolated and quantified in order to produce pools with equal amounts of total RNA (Table 1). Based on the estimated RNA concentrations of single samples, control pool C had a final concentration of 2.25 ng/μl and the RNA pool of high risk patients had a final concentration of 5 ng/μl. The exact final RNA concentrations and RNA quality of the generated pools was determined by an Agilent 2100 Bioanalyzer and Agilent RNA 6000 Pico Kit (Supplementary Figure 1). The electropherograms of pooled RNA samples indicated an appropriate length distribution of RNA. The concentrations were 1 ng/μl for group C pool and 2, 6 ng/μl for group HR. Both pools were used to produce four cDNA libraries with an RNA input of 10 ng. Different adapters with unique barcodes were added to individual libraries to enable multiplex sequencing. In order to obtain similar numbers of reads after sequencing, equal amounts of libraries were used to compose the final pool used for whole transcriptome analysis (Supplementary Table 2).

Next generation sequencing resulted in 255, 474, 790 reads for the final pool, which represents the depth of the collected transcriptome data. The quality of reads was analyzed using the software FastQC and was passed by all sequencing files, so that no additional corrections like trimming were necessary.

### Comparison of previously analyzed biomarkers in RT-qPCR and RNA-Seq

**Figure 1:**
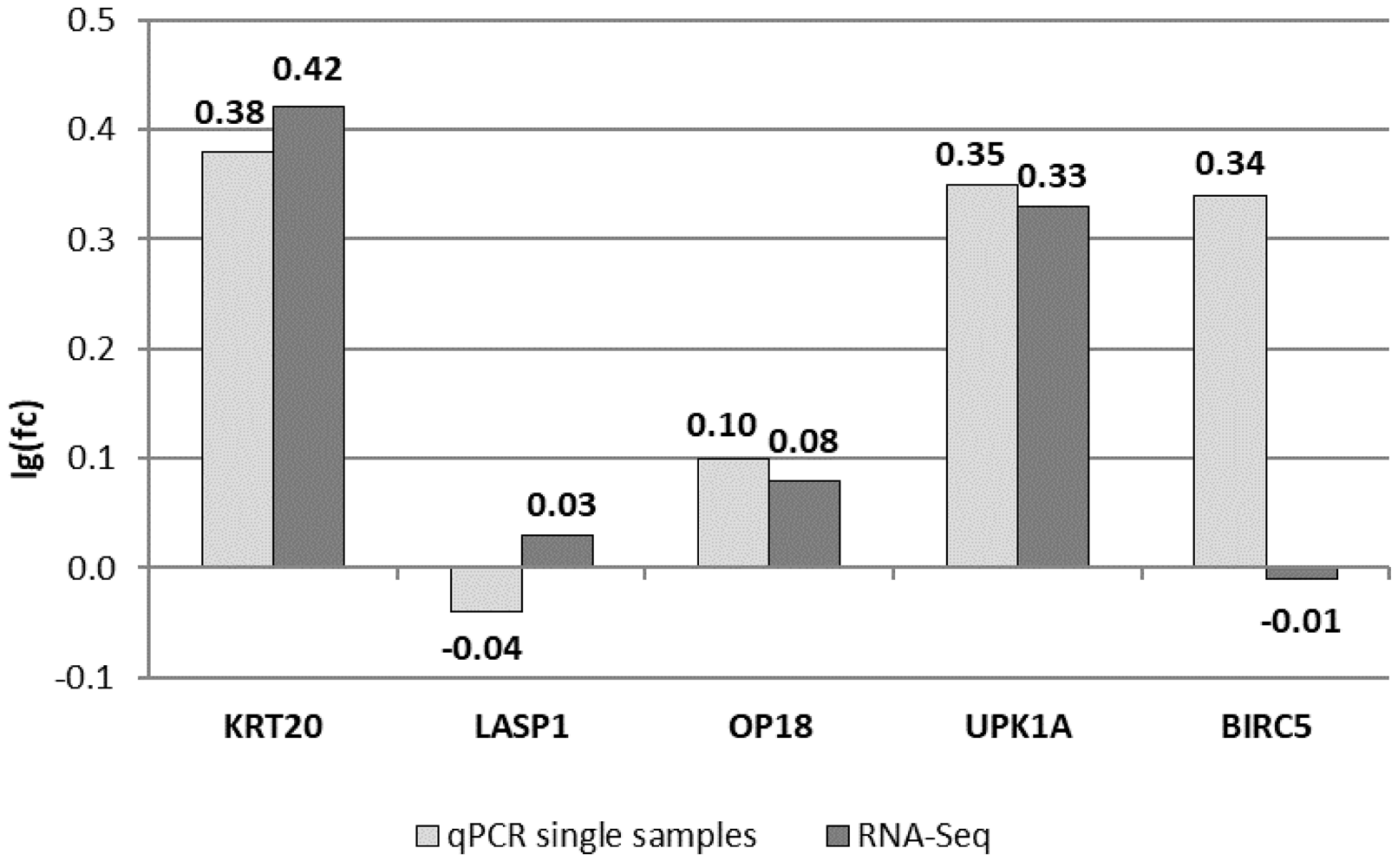
Comparing RT-qPCR results of single urine samples used for pools with RNA-Seq results. Shown are lg(fc) values between HR and C pools for five potential biomarkers, which were previously analyzed via RT-qPCR. All data are normalized to the expression of the 18S rRNA gene.

First, we tested whether the pooled samples of groups C and HR showed characteristics similar to recent findings for a limited set of potential tumor markers in single samples. To this end, we compared expression levels of the markers depicted in Table 1 and in Figure 1 that had been determined individually by RT-qPCR with the results of RNA-Seq measurements of urine pools (Figure 1). For the markers KRT20, LASP1, OP18 and UPK1A, the lg(fold change) (lg(fc)) values determined by either methodology were compatible. Only for BIRC5 we detected a discrepancy of differential gene expression of pools C and HR compared to the results of single samples for each group (Figure 1). In summary, we conclude that RNA-Seq essentially confirmed the marker measurements of single urine samples from control donors and high risk patients. Thus, we conclude that the transcriptome analysis of urine RNA with regard to the set of tumor marker candidates listed in Table 1 is valid and seems to be suitable for more detailed studies.

### Criteria for the selection of new potential biomarkers

In a first step, urine RNAs data identified by whole transcriptome analyses was ranked according to lg(fc) values comparing C and HR patient groups. Regarding the results of previously analyzed potential tumor markers (Figure 1), the threshold for new marker candidates was set to lg(fc) > 0.5 for transcripts with a higher expression or lg(fc) < -0.5 for transcripts with a lower expression of transcripts in high risk patients compared to healthy subjects. As the aim of this study was to find potential biomarkers with an improved sensitivity and specificity, all transcripts not fulfilling these criteria were excluded. For the search of more potent new marker candidates, we also included individual transcripts per kilobase million (TPM) values of group C and HR which should ideally not be smaller than 10 TPM for at least one value. Next, we looked at transcript support levels (TSL) provided by Ensembl, which give a hint on the real existence of bioinformatically predicted transcripts. Further, we considered potential or known biological information about the listed RNA species.

A more complex level of criteria is related to the predicted or known splicing pattern within a gene locus and to the exon/intron structure of individual transcripts. The more data we found on these characteristics, the more weight we put on selected transcripts. Finally, we set a length filter for selected transcripts of a minimal length of 150 nts. This was based on the requirement to be able to design appropriate amplicons for qPCR-based detection of selected RNA species.

### Classification of up- and down-regulated RNAs in pooled samples of the control and the high risk group

In the following, we describe classes of chosen RNA species of the control group and of the HR group according to the defined criteria. The defined categories include coding sequences (Supplementary Table 3A and 3B), non-coding transcripts (ncRNA) (Supplementary Table 3C and 3D), and circular RNAs (circRNA) (Supplementary Table 3E and 3F). We considered up-regulated transcripts as well as down-regulated transcripts between controls and HR samples for two reasons. First, a marker ratio of either one member of both groups has a higher chance to discriminate between both groups and, secondly, because a ratio of two makers is independent of absolute copy numbers. In the light of almost unmeasurable RNA concentrations in urine samples, this provides robustness to the detection of RNA markers.

For downregulated noncoding transcripts (Supplementary Table 3D), we were not able to identify RNAs matching the described criteria. Nevertheless, we showed the best marker candidates for this category. Furthermore, for circular transcripts (Supplementary Table 3E and 3F) we note that algorithms used in this study are not specifically for the identification of circRNA. In addition, no RNase R treatment of urine RNA was performed to ensure the detection of only circular RNA species. However, we include circRNA candidates in the lists shown in this study, because this class could contain RNA species with novel characteristics including their diagnostic value.

### Development of amplicons for RT-qPCR-based detection of potential tumor markers

RNA species for diagnostic purposes require robust and quantitative determination in urine samples. This can be achieved by reverse transcription of urine RNA followed by qPCR-based methodology. Thus, we developed and thoroughly characterised marker-specific pairs of PCR primers for PCR reactions for selected and promising markers described in this study. Because of very limited RNA amounts in human urine and the lack of sufficient patient material, we performed initial studies in the use of a cell culture model system which we recently described [32]. In this dual model, the human control group C is represented by RT-4 cells which were established from a G1 stage urinary bladder carcinoma [33]. The human HR group and more progressed tumors are reflected by ECV-304 cells which had been derived from a G3 bladder carcinoma [34, 35].

A thorough analysis of transcripts that may serve as tumor marker candidates indicates that the design of amplicons for the PCR-based measurement has to consider the complex potential composition of transcripts expressed from a specific locus as well as the differential expression levels (lg(fc)) and the TSL values. Instructive examples are shown for C-X-C motif chemokine ligand 8 (CXCL8)-, ribosomal protein S27 (RPS27)- and ribosomal protein L23a (RPL23A)-specific RNAs (Table 2).

**Table 2:** Transcripts of (A) CXCL8, (B) RPS27 and (C) RPL23A

**(A) CXCL8 (C-X-C motif chemokine ligand 8)**							
**Transcript ID**	**Name**	**length [kb]**	**HR [TPM]**	**C [TPM]**	**log(HR/C)**	**TSL**	**Biotype**
**ENST00000307407**	**CXCL8-201**	**1.705**	**618.357**	**148.984**	**0.618**	**1**	**Ri**
**ENST00000401931**	**CXCL8-202**	**0.700**	**365.908**	**86.693**	**0.625**	**1**	**Pc**
**ENST00000483500**	**CXCL8-203**	**0.599**	**162.685**	**45.813**	**0.550**	**2**	**Pc**
**∑**	**-**	**-**	**382.32**	**93.83**	**0.61**	**-**	**-**

**(B) RPS27 (ribosomal protein S27)**							
**Transcript ID**	**Name**	**length [kb]**	**HR [TPM]**	**C [TPM]**	**log(HR/C)**	**TSL**	**Biotype**
**ENST00000368567**	**RPS27-201**	**0.359**	**779.327**	**258.917**	**0.479**	**1**	**Pc**
ENST00000493224	RPS27-203	0.573	44.177	14.822	0.474	2	Ri
ENST00000477151	RPS27-202	0.496	50.634	18.841	0.429	2	Ri

**(C) RPL23A (ribosomal protein L23a)**							
**Transcript ID**	**Name**	**length [kb]**	**HR [TPM]**	**C [TPM]**	**log(HR/C)**	**TSL**	**Biotype**
**ENST00000422514**	**RPL23A-204**	**0.970**	**127.601**	**38.520**	**0.520**	**1**	**Pc**
ENST00000496182	RPL23A-206	0.636	35.841	11.507	0.493	3	Pc
**ENST00000355731**	**RPL23A-201**	**0.587**	**36.773**	**11.417**	**0.508**	**5**	**Pc**
ENST00000394935	RPL23A-202	0.580	36.805	10.828	0.531	5	Pc
ENST00000472628	RPL23A-205	0.639	37.069	13.994	0.423	1	Pc
ENST00000582736	RPL23A-209	0.595	151.180	49.356	0.486	1	Ri
ENST00000394938	RPL23A-203	0.688	30.541	10.953	0.445	2	Pc
ENST00000578181	RPL23A-207	0.593	33.195	11.832	0.448	2	Pc
ENST00000580755	RPL23A-208	0.412	13.270	9.550	0.143	2	Ri
**∑**	**-**	**-**	**82.19**	**24.97**	**0.52**	**-**	**-**

All RNA species of CXCL8, RPS27 and RPL23A detected in the transcriptome data were listed and TPM and TSL values as well as the exon-intron structure of individual transcripts was analyzed. Primer pairs were designed according to material and methods and RNA species were checked for a specific amplification. Only transcripts marked in bold letters are targeted by both primers and result in a finally calculated lg(fc) value for a potential new biomarker. (Legend: Protein coding (Pc), Retained Intron (Ri)).

A list of RNA marker loci and major characteristics of transcripts is shown in Table 2. For all three identified CXCL8-specific splice variants we determined promising TSL and TPM values, which should give rise to a final lg(fc) value of 0.61 for all CXCL8 RNA species (Table 2A). For RPS27 (Table 2B), the primer pair with an unequivocal and strong PCR signal in cell lines was targeting only the most promising transcript RPS27-201 with an lg(fc) value of 0.48, a TSL value of 1 and the highest TPM levels. In contrast, the splicing pattern for RPL23A (Table 2C) was more complex. We found a large set of RNA species with different TSL values for this gene locus. The finally selected amplicons recognise only the two transcripts RPL23A-204 and RPL23A-201. Regardless of the existence of the splice variant RPL23A-202 with a TSL value of 5 the calculated lg(fc) value for both transcripts is 0.52.

It should be noted that the filters we used to identify promising marker sequences seems to be very stringent. For example, for the most promising candidate Glycin-N-Acetyltransferase (GLYATL1)-206 with a lg(fc) value of 1.624 (Supplementary Table 3A) we could not detect a PCR signal in the use of either amplicon and the two urinary bladder cancer cell lines. Similarly, the most promising splicing variant of the potential tumor marker with the lowest log(fc) value in the high risk group, i.e., Zinc finger protein 382 (ZNF382)-208 (Supplementary Table 3B) was excluded, as was Ryanodine receptor 3 (RYR3)-227, which was not detectable in ECV-304 cells nor in RT-4 cells. We cannot tell whether this is due to low TSL values of 4 for ZNF382-208 and 5 for RYR3-227. These examples demonstrate the complexity of developing a specific and sensitive PCR amplicon for a potential marker transcript with regard to the composition of transcripts expressed from a specific locus, the differential expression levels, and the TSL values.

### Amplicons for the detection of linear and circular S100A6-specific transcripts and for TRAM1-specific transcripts

Both hits, the potential biomarkers S100 Calcium Binding Protein 6 (S100A6) and translocation associated membrane protein 1 (TRAM1) show an impressive correlation of linear and circular transcripts of these gene loci with the health status of donors (Table 3). While most of linear TRAM1-specific RNA species show a minor under-expression in urine pooled RNA of HR patients, all circular TRAM1 transcripts indicate a clear and high reduction of gene expression in the HR group compared to the control group (Table 3A). Hence, divergent primers for the detection of only circular transcripts were developed to target all circular TRAM1 splicing variants resulting in a promising lg(fc) value of –1.26. Convergent primer pairs for the amplification of linear sequence segments were designed to match with linear TRAM1 RNA species with negative lg(fc) values. It is noteworthy that TRAM1-203 with the lowest lg(fc) value of –1.09 is related to a TSL of 5. Thus, the existence of this variant is uncertain. In summary, transcripts of the TRAM1 gene locus represent the most promising candidates for potent tumor markers with decreased gene expression in high risk patients. For a robust discrimination between healthy individuals and patients with bladder cancer, it would be beneficial to find marker candidates with inverse changes of gene expression, as explained above.

**Table 3:** Linear and circular transcripts of TRAM1 and S100A6

**(A) TRAM1 (translocation associated membrane protein 1)**					
**Transcript ID**	**Name**	**length [kb]**	**HR [TPM]**	**C [TPM]**	**log(HR/C)**
**hsa_circ_0084758**	**TRAM1**	**0.176**	**0.66**	**41.33**	**–1.80**
**hsa_circ_0084756**	**TRAM1**	**0.383**	**0.81**	**21.56**	**–1.43**
**hsa_circ_0084757**	**TRAM1**	**0.447**	**0.98**	**18.51**	**–1.28**
**hsa_circ_0084759**	**TRAM1**	**0.422**	**3.08**	**20.04**	**–0.81**
**∑**	**-**	**-**	**1.38**	**25.36**	**–1.26**

**Transcript ID**	**Name**	**length [kb]**	**HR [TPM]**	**C [TPM]**	**log(HR/C)**	**TSL**	**Biotype**
**ENST00000520700**	**TRAM1-203**	**0.542**	**1.630**	**20.115**	**–1.091**	**5**	**Pt**
**ENST00000521425**	**TRAM1-205**	**3.394**	**5.126**	**8.141**	**–0.201**	**2**	**Pc**
**ENST00000262213**	**TRAM1-201**	**2.785**	**6.077**	**8.431**	**–0.142**	**1**	**Pc**
ENST00000521049	TRAM1-204	0.872	3.700	2.805	0.120	5	Pt
ENST00000518678	TRAM1-202	0.535	4.339	3.043	0.154	4	Pc
**∑**	**-**	**-**	**4.28**	**12.23**	**–0.46**	**-**	**-**

**(B) S100A6 (S100 Calcium Binding Protein 6)**					
**Transcript ID**	**Name**	**length [kb]**	**HR [TPM]**	**C [TPM]**	**log(HR/C)**
**hsa_circ_0014225**	**S100A6**	**0.390**	**391.17**	**102.25**	**0.58**
**hsa_circ_0014226**	**S100A6**	**0.683**	**314.28**	**87.06**	**0.56**
hsa_circ_0014224	S100A6	0.231	105.43	29.96	0.55
hsa_circ_0014227	S100A6	0.452	21.51	7.32	0.47
**∑**	**-**	**-**	**352.73**	**94.66**	**0.57**

**Transcript ID**	**Name**	**length [kb]**	**HR [TPM]**	**C [TPM]**	**log(HR/C)**	**TSL**	**Biotype**
**ENST00000496817**	**S100A6-205**	**0.673**	**79.051**	**19.216**	**0.614**	**2**	**Pc**
ENST00000462951	S100A6-204	0.311	10.455	4.698	0.347	1	Ri
**ENST00000368720**	**S100A6-202**	**0.673**	**70.632**	**17.232**	**0.613**	**3**	**Pc**
**ENST00000368719**	**S100A6-201**	**0.665**	**120.508**	**28.035**	**0.633**	**1**	**Pc**
ENST00000462776	S100A6-203	0.479	13.011	4.239	0.487	2	Pc
**∑**	**-**	**-**	**90.06**	**21.49**	**0.62**	**-**	**-**

All RNA species of TRAM1 and S100A6 detected in the transcriptome data were listed and TPM and TSL values as well as the exon-intron structure of individual transcripts was analyzed. Primer pairs were designed according to material and methods and RNA species were checked for a specific amplification. Only transcripts marked in bold letters are targeted by both primers and result in a finally calculated lg(fc) value for a potential new biomarker. (Legend: Protein coding (Pc), Retained Intron (Ri), Processed transcript (Pt)).

This fits well with the second potential biomarker S100A6 with a correlation of increased linear and circular transcripts in urine pools of patients with high risk BCa (Table 3B). In contrast to the TRAM1 system, the design of divergent primers targeting all circRNAs of S100A6 was not possible. Thus, primer pairs were developed to detect only the two RNA species indicating the highest lg(fc) values. For linear S100A6 transcripts the two splicing variants with the lowest differential gene expression could be excluded. This approach would lead to theoretically calculated log(fc) values of approximately 0.6 for the PCR amplicons of both RNA classes. This suggests S100A6 transcripts as another promising candidate for tumor markers overexpressed in the HR group.

### Analysis of differential gene expression of potential tumor markers in urinary bladder carcinoma cell lines

Finally, we studied differential gene expression of selected tumor markers in the dual cell culture model system. Differences in gene expression of RT-4 and ECV-304 cells could give a first hint on potential RNA markers in urine of BCa patients. Therefore, we validated the PCR amplicons developed in this study in both cell lines and compared calculated lg(fc) values with the data of transcripts in urine samples of patients (Table 4).

**Table 4:** Differences in gene expression of selected markers in ECV-304 and RT-4 cells compared to the results of urine pools C and HR of RNA-Seq

Transcript	HR [TPM]	C [TPM]	lg(HR/C)	ECV-304	RT-4	lg(ECV-304/ RT-4)
CXCL8	382.32	93.83	0.61	111.86	181.13	-0.21
RPS27	395.04	133.20	0.47	149.51	81.14	0.27
RPL23A	82.19	24.97	0.52	327.93	383.14	-0.07
TRAM1	4.28	12.23	-0.46	35.83	143.36	-0.60
S100A6	90.06	21.49	0.62	1027.96	281.88	0.56

Shown are the TPM and lg(fc) values of the high risk (HR) and the control (C) patient pool for five potential biomarkers, which were previously analyzed via RNA-Seq. These selected marker candidates were also analyzed in bladder cancer cell lines (ECV-304 and RT-4) via RT-qPCR (see Tables 2 and 3). All data was normalized to the expression of 18S rRNA and used for calculation of lg(fc) values.

For marker candidates CXCL8 and RPL23A, lg(fc) values are not compatible between the cell culture models versus patient samples. Conversely, for transcripts of RPS27, TRAM1, and S100A6 these values are consistent between pooled urine RNA samples of control and HR patients and RT-4 and ECV-304 cells, respectively. TRAM1 transcripts show decreased expression in urine of high risk BCa patients and in ECV-304 cells which represent the G3 cancer stage. As a potential biomarker with a higher expression in G3 cells and in HR urine pool, we identified S100A6 transcripts. These RNAs exhibit a clear differential gene expression and, thus, they were selected for calculation of RNA ratios (Table 5).

**Table 5:** RNA ratio of TRAM1 and S100A6 transcripts in ECV-304 and RT-4 cells compared to the results of urine pools C and HR of RNA-Seq

RNA ratio of S100A6/ TRAM1 transcripts					
ECV-304	RT-4	lg(ECV-304/ RT-4)	HR	C	lg(HR/C)
28.69	1.97	1.16	21.04	1.76	1.08

RNA ratios were calculated from TPM values of high risk and control patient pool and from copies of bladder carcinoma cell lines shown in Table 4.

The ratio of S100A6/TRAM1 transcript signals showed a high consistency between cell culture cells and transcriptome analyses of urine pools of human donors. Based on these findings a discrimination of healthy individuals and HR patients seems to be feasible by a factor of 10. Hence, the S100A6/TRAM1 ratio seems to be a significant and highly promising marker for the non-invasive diagnosis of bladder cancer based on urine RNA. With regard to this finding, it might be even more promising to study circular forms of S100A6-specific- and TRAM1-specific transcripts.

## DISCUSSION

Whole transcriptome analysis of urine RNA from as little as a few milliliter urine is feasible. This study describes a significant extension of the concept of non-invasive diagnosis of bladder cancer in the use of RNA markers. This molecular concept is attractive for early tumor diagnosis because in case of aberrant gene expression which is linked with malignant cell growth, RNA is the first gene product to be detected even before gene products or their metabolic effects are detectable. It is noteworthy that the technology is robust and reliable. This includes the collection of urine samples at small volumes, the stabilization of RNA, shipping, storage, and the quantitative detection of RNA transcripts via RT-qPCR. All steps are based on established standard operation procedures [21, 32, 36]. While the proof-of-concept has been reported manifold in the past including potential bladder cancer-specific tumor markers [19, 37, 38], it remains open, what kind of gold standard in this methodological category will be identified and whether RNA markers can support or substitute the current gold standard, i.e. cystoscopy.

**Figure 2:**
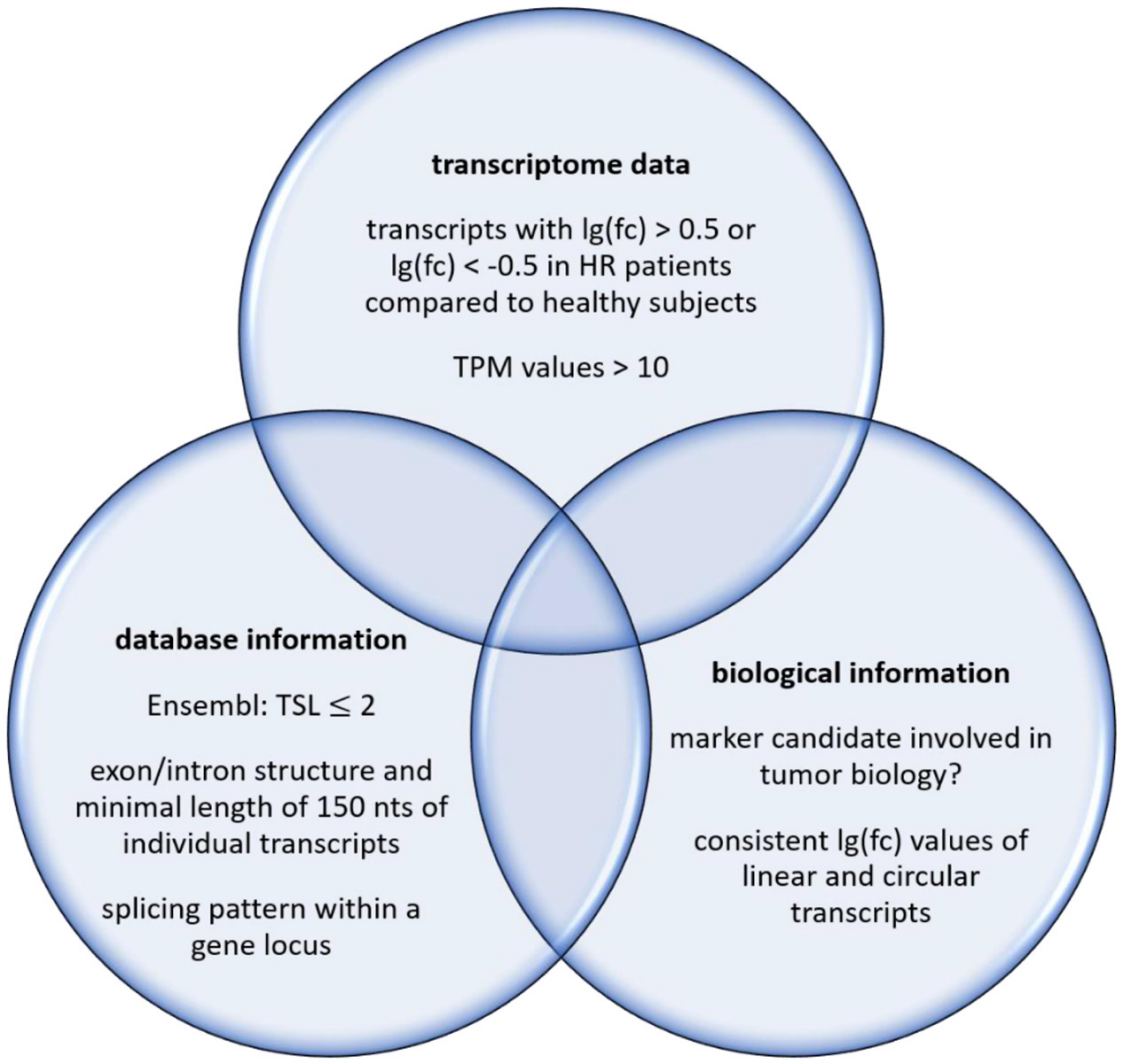
Filters for a systematic search of RNA-based markers.

With regard to these considerations, we performed a systematic search for RNA-based markers by a whole transcriptome approach. The underlying rational is shown schematically in Figure 2. Briefly, we considered transcriptome data with defined threshold for differential transcript abundances, characteristics recorded by database, and known or suspected biological roles of potential hits. The number of RNA sequences and gene loci, respectively seems to be large enough in order to be able to identify transcripts that differ substantially between the healthy state and bladder cancer patients. Subsequent to the detailed analyses described here, this study suggests considering RNA-based tumor markers for this cancer type. It also suggests investigating in more detail and depth rationally identified marker candidates.

Here we look at RNA expression data derived from different methodological approaches. Hence, it is a central issue of this study to study whether there is compatibility of marker candidates derived from deep sequencing of pools of patient samples versus individual RT-qPCR-based data of individual marker candidates (Figure 1). This finding depicted in Figure 1 suggests to analyze deep transcriptome data in more detail in order to identify substantially improved RNA markers for bladder cancer. This procedure opens a systematic and rational approach for marker identification which is independent of prior knowledge in the area of tumor biology and, thus, it is almost completely unbiased.

One of the most remarkable findings of this study is related to the ratio of S100A6/TRAM1. This ratio seems to represent an improved potential marker for human bladder cancer (Table 5) and it is also found in the dual cell culture model consisting of RT-4 cells and ECV-304 cells. Taken together, these observations provide a coherent picture of the disease-related changes of gene expression of S100A6 and TRAM1 suggesting to analyze this ratio in a large diagnostic study. However, a larger number of marker candidates was also described in this work (Tables 2 and 3) and these lists can be extended by a ranked list of more transcripts.

While all of these hits focus on conventional linear transcripts we also noted experimental evidence supporting the existence of circular RNAs that seem to be able to monitor differences between the healthy state and the cancer state. This is reflected by a number of recent studies describing a link between circular RNA and cancer [30, 31]. However, such evidence derived from *in silico* analyses does not necessarily proof that such circRNAs exist in reality. A set of experimental evidence is necessary to enough strongly verify their existence. Methods include PCR-based approaches that can detect backsplicing sites, sequencing of cloned backsplicing sites, and independent orthogonal tests such as RNaseH-based cleavage analyses. However, such assays go beyond the scope of this study although this is one promising way to proceed in order to identify new and potent RNA markers.

## MATERIALS AND METHODS

### Clinical samples

This study was approved by the local ethical committee of the Universität zu Lübeck. All urine samples were obtained with written consent of the participants. All bladder cancer patients were classified according to the World Health Organization 2004 grading and risk system. For the investigation of urinary RNA, spontaneously voided urine of donors (healthy individuals, *n* = 47; high risk (HR) patients, *n* = 66) was collected and stabilized immediately with one volume of a lysis buffer (6 mol/l guanidinium isothiocyanate, 0.05 mol/l sodium acetate, and 0.5% N-lauroylsarcosine) as described recently [36]. Stabilized urine samples were frozen in liquid nitrogen and stored at –80°C until RNA preparation. For details of urine sampling see Supplementary Table 1.

### Isolation of RNA from urine samples

To isolate RNA from human urine samples, the RNeasy Midi Kit (QIAGEN, Hilden, Germany) was used with minor modifications. Briefly, stabilized urine samples were thawed slowly and adjusted to pH 7.0 by adding 1 M HEPES buffer. Instead of using RLT buffer, recommended volumes of 70% ethanol and mercaptoethanol were added directly to the samples. Subsequent steps were performed following the manufacturer’s instructions. RNA samples were eluted in 320 μl of RNase-free water, lyophylized, and resuspended in 16 μl of RNase-free water. Samples were stored at –80°C.

### RNA quantification and quality assessment

RNA samples were quantified using a NanoDrop ND-1000 spectrophotometer (Thermo Fisher Scientific, Waltham, MA, USA). To measure concentration and integrity of pooled urinary RNA, the Agilent 2100 Bioanalyzer was used in combination with the Agilent RNA 6000 Pico Kit (Agilent Technologies, Santa Clara, CA, USA) and the manufacturer’s instructions were followed.

### Synthesis, purification and quantification of double-stranded cDNA libraries

For sequencing on Illumina platforms, cDNA libraries were synthesized using the SMARTer Stranded Total RNA-Seq Kit – Pico Input Mammalian (TaKaRa Bio Inc., Kusatsu, prefecture Shiga, Japan). Briefly, 10 ng of total RNA was fragmented according to manufacturer’s instructions and used for first-strand synthesis followed by addition of Illumina adapters. Ribosomal cDNA was depleted and the final double-stranded cDNA library was amplified via PCR according to manufacturer’s instructions. Before depletion of ribosomal cDNA and after PCR amplification, the double-stranded cDNA libraries were purified using the Agencourt AMPure XP PCR purification system (Beckman Coulter, Brea, CA, USA). The first purification step was performed according to the manufacturer´s instructions. The second purification step contained minor modifications. Briefly, the amount of beads was increased to 100 μl volume and the cDNA was eluted in 20 μl elution buffer. To quantify the double-stranded cDNA libraries, the Qubit dsDNA HS Assay Kit and the Qubit Fluorometer (Thermo Fisher Scientific, Waltham, MA, USA) were used.

### RNA sequencing

Previously synthesized double-stranded cDNA libraries were sequenced by GATC Biotech AG (GATC Biotech AG, Konstanz, Germany). The Illumina platform was used with a paired-end (PE) mode and a read length of 125 nucleotides.

### Data quality control

To check the quality of reads provided by GATC Biotech AG, the software FastQC (https://www.bioinformatics.babraham.ac.uk/projects/fastqc/) by Simon Andrews at Babraham Bioinformatics was used.

### Data analysis

Codes for data analysis were written in the UNIX command line. Briefly, three different reference data sets were downloaded: the cDNA and ncRNA databases provided by Ensembl [39] and the circRNA database provided by circBase [40]. Databases were indexed to accelerate the mapping process and read mapping was performed using the bwa software [41]. Resulting mapped reads were recorded and normalized to TPMs (transcripts per million) to eliminate biases introduced by transcript length and sequencing depth [42]. For analysis of differential gene expression of marker candidates, lg(fold change) values of expression levels were calculated and compared for pooled samples of C and HR patient group.

### cDNA synthesis

Reverse transcription was performed using the SuperScript III First-Strand Synthesis Kit (Thermo Fisher Scientific, Waltham, MA, USA) in a total volume of 20 μl containing RNA extract and 300 ng of random hexamer primer (Invitrogen, Paisley, UK). Manufacturer’s instructions were followed using the 16 μl of resuspended urinary RNA with an RNA input of 7.5 μl for the RT sample and 7.5 μl for the non-RT control sample. It should be noted that RNA concentrations in urine are extremely low and Nanodrop-based measurements of UV absorption cannot be used. However, the length distribution of urine RNA seems to be suitable [16] and RT-qPCR-based detection is possible. Therefore, urinary RT and non-RT samples were diluted 1:16 and 1:160 for detection of marker candidates and 18S rRNA in qPCR, respectively.

For cDNA synthesis of cellular RNA, RevertAid First Strand cDNA Synthesis Kit (Thermo Fisher Scientific, Waltham, MA, USA) was used and manufacturer’s instructions were followed with 2 μg RNA input of bladder cancer cell lines. For all non-RT control reactions, nuclease-free water was added instead of solutions of RNaseOut or RiboLock RNase Inhibitor and reverse transcriptase.

### Quantitative PCR (qPCR)

qPCR was performed using SYBR green and TaqMan Systems in the 384-well plate format. Primer concentrations was 200 nM and concentrations of TaqMan probes was 250 nM for a 10 μl total reaction volume with 4 μl template of RT or non-RT sample and 5 μl of SYBR green or TaqMan Master Mix. SYBR green reactions were performed with a SYBR Select Master Mix (Thermo Fisher Scientific, Waltham, MA, USA.). The thermal cycler 7900HT (Applied Biosystems, Foster City, CA, USA) conditions were 50°C for 120 seconds, 95°C for 120 seconds, and 40 cycles consisting of 95°C for 15 seconds, and 60°C for 60 seconds. Melting curve analysis was performed. For the TaqMan system, TaqMan Universal Master Mix II (Applied Biosystems, Foster City, CA, USA) was used with thermocycler conditions of 50°C for 120 seconds, 95°C for 600 seconds, and 40 cycles consisting of 95°C for 15 seconds and 60°C for 60 seconds. Samples were measured in quadruplicates and negative controls without reverse transcriptase and RNaseOut or without template were included. Data analysis was performed via the SDS 2.1 software (Applied Biosystems, Foster City, CA, USA). The RNA level of bladder cancer cell lines was normalized to the levels of endogenous 18S ribosomal RNA which served as an internal control. Markers of urinary RNA were quantified and normalized according to serial 10-fold dilutions of plasmid standards.

### Design of primer pairs and TaqMan probes

Primer pairs and TaqMan probes are shown in Supplementary Table 4. Convergent primer pairs for detection of linear sequences of transcripts were designed using NCBI Primer-BLAST (https://www.ncbi.nlm.nih.gov/tools/primer-blast/) with a melting temperature of 57–63°C (optimum 60°C, maximal T_m_ difference 3°C). The PCR product size was adjusted to a range of 70–150 nucleotides. Three types of primer pairs were created for each transcript: Primer pairs in the same exon, primer pairs separated by one intron, and primer pairs with one primer spanning the exon-exon junction sequence. In the last step, primer pairs were checked for specificity of amplification of the target transcripts and problematic secondary structures as well as self-complementarity were excluded. Divergent primer pairs for the detection of circular transcripts were designed according to “Circular RNA Interactome” [43], which was used to identify back-splicing junction sequences of a circular RNA. This sequence was inserted into NCBI Primer-BLAST (https://blast.ncbi.nlm.nih.gov/Blast.cgi) and the design of primer pairs was performed as described above. TaqMan probes were designed using Primer Express software version 2.0 (Applied Biosystems, Foster City, CA, USA) or Primer3 software (Steve Rozen, Whitehead Institute for Biomedical Research, Cambridge, UK). Primer pairs and TaqMan probes were purchased from Metabion (Planegg/ Steinkirchen, Germany) and Eurogentec (Seraing, Belgium).

### Cell culture

The human urinary BCa cell line ECV-304 was cultivated in Medium 199 (with HEPES buffer + Earle’s salts) (PAA, Pasching, Austria) containing 10% (vol/vol) fetal calf serum (PAA, Pasching, Austria). ECV-304 was originally established from an invasive, G3 BCa of an 82 years old Swedish female patient with a mutant p53 in 1970. It is a defined derivative of T24 [34, 35] which we obtained from the DSMZ (Deutsche Sammlung von Mikroorganismen und Zellkulturen, Braunschweig, Germany), a repository for microorganism and cell lines. Cell identity was confirmed by DNA profiling by the DSMZ. RT-4 cells [33] were cultivated in RPMI 1640 medium (PAA, Pasching, Austria) supplemented with 10% (vol/vol) fetal calf serum. RT-4 cells were used as an *in vitro* model for differentiated G1 BCa. Both cell lines were cultivated without antibiotics at 37°C and 5% CO2 in a humidified incubator. For quantification of PCR amplicons, cells were washed twice with PBS, harvested and lysed using QIAzol Lysis Reagent (Qiagen, Hilden, Germany) according to manufacturer’s instructions. The total cellular RNA was isolated using phenol-chloroform extraction followed by ethanol precipitation.

## SUPPLEMENTARY MATERIALS



## Abbreviations

BCa: Human bladder cancer
circRNA: circular RNA
ncRNA: non-coding transcripts
lg(fc): lg(fold change)
BIRC5: Baculoviral IAP repeat-containing protein 5
LASP1: LIM and SH3 protein
KRT20: Keratin 20
OP18: Oncoprotein 18
UPK1A: Uroplakin 1A
CXCL8: C-X-C motif chemokine ligand 8
RPS27: ribosomal protein S27
RPL23A: ribosomal protein L23a
TRAM1: translocation associated membrane protein 1
S100A6: S100 Calcium Binding Protein 6
GLYATL: Glycin-N-Acetyltransferase
ZNF382: Zinc finger protein 382
RYR3: Ryanodine receptor 3
TPM: transcripts per kilobase million
TSL: transcript support level
HR: high risk patient group
C: control patient group
