# Supplementary material for: Transcriptome analyses of urine RNA reveal tumor markers for human bladder cancer: validated amplicons for RT-qPCR-based detection
Source: Oncotarget. 2021 May 11;12(10):1011–23. doi: 10.18632/oncotarget.27954 (PMC8121610; doi:10.18632/oncotarget.27954)
Supplement: Supplementary file 1 [file oncotarget-12-1011-s001.pdf]

# Transcriptome analyses of urine RNA reveal tumor markers for human bladder cancer: validated amplicons for RT-qPCR-based detection

## SUPPLEMENTARY MATERIALS

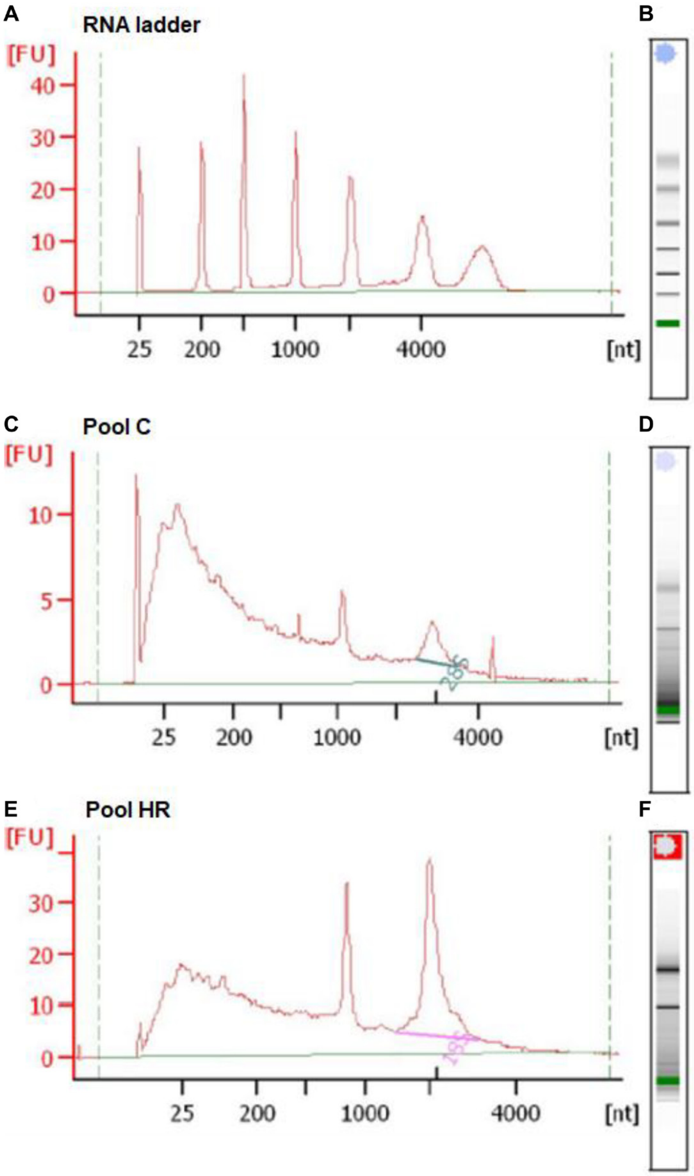


**Supplementary Figure 1: Analysis of RNA pools reveals high RNA degradation.** The Agilent RNA 6000 Pico Kit was used to analyze the integrity of the RNA in pools C and HR. (**A**, **C** and **E**) show the electropherograms of a control RNA ladder, pool C and pool HR, respectively. (**B**, **D** and **F**) show the gel images of a control RNA ladder, pool C and pool HR respectively. The absence of distinct peaks (C and E) and bands (D and F) in comparison to the control RNA ladder indicate significant RNA degradation.

## Supplementary Table 1: Clinical characteristics of patients

| Pool | Patient Number | Sex | Grade | Risk | Stage | Recurrence |
|---|---|---|---|---|---|---|
| | 48 | f | - | - | - | - |
| | 50 | m | - | - | - | + |
| | 80 | m | - | - | - | + |
| **C** | 138 | m | - | - | - | + |
| | 160 | m | - | - | - | - |
| | 166 | f | - | - | - | + |
| | 170 | m | - | - | - | - |
| | 41 | m | G3, HG | HR | Ta | - |
| | 46 | m | G2, HG | HR | Ta | - |
| | 107 | f | G2, HG | HR | Ta | + |
| **HR** | 128 | m | G3, HG | HR | Ta | - |
| | 159 | m | G3, HG | HR | T2-4 | - |
| | 164 | m | G3, HG | HR | Ta | + |
| | 183 | m | HG | HR | T2-4 | - |

Description: f = female, m = male, C = control group, HR = high risk, HG = high grade, Ta = noninvasive papillary BCa, T2-4 = invasive BCa, G2/G3 = grading of BCa.

## Supplementary Table 2: Final cDNA pool composition for sequencing

| Library # | Content | Concentration [ng/µl] | Contribution in final pool [%] |
|---|---|---|---|
| **1** | C | 24.4 | 21 |
| **2** | C | 16.5 | 32 |
| **3** | HR | 19.8 | 26 |
| **4** | HR | 23.6 | 21 |

Shown are content, concentration and contribution in final pool for cDNA libraries 1–4.

**Supplementary Table 3: Classification of up- and down-regulated RNAs in pooled samples of the control group C and the HR group**

**(A) Up-regulated coding transcripts in urine of high risk patients compared to healthy subjects**

| Transcript ID | Name | length [kb] | HR [TPM] | C [TPM] | log(HR/C) | TSL | Biotype |
|---|---|---|---|---|---|---|---|
| ENST00000525608 | GLYATL1-206 | 0.573 | 37.616 | 0.895 | 1.624 | 2 | Pc |
| ENST00000320868 | HBA1-201 | 0.577 | 163.828 | 8.190 | 1.301 | 1 | Pc |
| ENST00000472694 | HBA1-203 | 0.674 | 42.293 | 2.529 | 1.223 | 1 | Ri |
| ENST00000487791 | HBA1-204 | 0.410 | 12.091 | 1.361 | 0.948 | 1 | Ri |
| ENST00000397797 | HBA1-202 | 0.504 | 58.325 | 2.586 | 1.353 | 2 | Pc |
| ENST00000397806 | HBA2-201 | 0.513 | 44.780 | 2.261 | 1.297 | 2 | Pc |
| ENST00000482565 | HBA2-203 | 0.640 | 24.005 | 2.314 | 1.016 | 1 | Pc |
| ENST00000484216 | HBA2-204 | 0.403 | 12.415 | 2.224 | 0.747 | 1 | Pc |
| ENST00000335295 | HBB-201 | 0.628 | 128.769 | 7.801 | 1.218 | 1 | Pc |
| ENST00000485743 | HBB-204 | 0.680 | 24.824 | 7.685 | 0.509 | 1 | Pc |
| ENST00000475226 | HBB-203 | 0.319 | 29.203 | 1.980 | 1.169 | 2 | Ri |
| ENST00000633227 | HBB-205 | 0.609 | 94.092 | 5.025 | 1.272 | 3 | NMD |
| ENST00000307407 | CXCL8-201 | 1.705 | 618.357 | 148.984 | 0.618 | 1 | Ri |
| ENST00000401931 | CXCL8-202 | 0.700 | 365.908 | 86.693 | 0.625 | 1 | Pc |
| ENST00000483500 | CXCL8-203 | 0.599 | 162.685 | 45.813 | 0.550 | 2 | Pc |
| ENST00000466565 | MBNL1-214 | 1.096 | 25.787 | 2.723 | 0.976 | 1 | Pc |
| ENST00000496817 | S100A6-205 | 0.673 | 79.051 | 19.216 | 0.614 | 2 | Pc |
| ENST00000368720 | S100A6-202 | 0.673 | 70.632 | 17.232 | 0.613 | 3 | Pc |
| ENST00000368719 | S100A6-201 | 0.665 | 120.508 | 28.035 | 0.633 | 1 | Pc |
| ENST00000462776 | S100A6-203 | 0.479 | 13.011 | 4.239 | 0.487 | 2 | Pc |
| ENST00000422514 | RPL23A-204 | 0.970 | 127.601 | 38.520 | 0.520 | 1 | Pc |
| ENST00000496182 | RPL23A-206 | 0.636 | 35.841 | 11.507 | 0.493 | 3 | Pc |
| ENST00000355731 | RPL23A-201 | 0.587 | 36.773 | 11.417 | 0.508 | 5 | Pc |
| ENST00000394935 | RPL23A-202 | 0.580 | 36.805 | 10.828 | 0.531 | 5 | Pc |
| ENST00000582736 | RPL23A-209 | 0.595 | 151.180 | 49.356 | 0.486 | 1 | Ri |
| ENST00000368567 | RPS27-201 | 0.359 | 779.327 | 258.917 | 0.479 | 1 | Pc |
| ENST00000493224 | RPS27-203 | 0.573 | 44.177 | 14.822 | 0.474 | 2 | Ri |

**(B) Down-regulated coding transcripts in urine of high risk patients compared to healthy subjects**

| Transcript ID | Name | length [kb] | HR [TPM] | C [TPM] | log(HR/C) | TSL | Biotype |
|---|---|---|---|---|---|---|---|
| ENST00000585467 | ZNF382-208 | 0.550 | 17.540 | 293.821 | -1.224 | 4 | Pt |
| ENST00000637522 | RYR3-227 | 0.137 | 0.236 | 3.701 | -1.196 | 5 | Pt |
| ENST00000390499 | TRAJ38-201 | 0.062 | 0.520 | 6.923 | -1.124 | NA | TR J gene |
| ENST00000520700 | TRAM1-203 | 0.542 | 1.630 | 20.115 | -1.091 | 5 | Pt |

**(C) Up-regulated noncoding transcripts in urine of high risk patients compared to healthy subjects**

| Transcript ID | Name | length [kb] | HR [TPM] | C [TPM] | log(HR/C) | TSL | Biotype |
|---|---|---|---|---|---|---|---|
| ENST00000414790 | H19-203 | 2.348 | 1.47 | 7.97 | 0.73 | 1 | LncRNA |
| ENST00000412788 | H19-202 | 2.281 | 1.24 | 7.36 | 0.77 | 1 | LncRNA |
| ENST00000439725 | H19-209 | 1.929 | 0.90 | 6.42 | 0.85 | 2 | LncRNA |
| ENST00000411861 | H19-201 | 1.771 | 1.30 | 5.35 | 0.61 | 2 | Ri |
| ENST00000417089 | H19-204 | 1.090 | 0.69 | 5.56 | 0.90 | 5 | LncRNA |
| ENST00000431095 | H19-207 | 0.944 | 0.87 | 6.31 | 0.86 | 5 | Ri |

| | | | | | | | |
|---|---|---|---|---|---|---|---|
| ENST00000428066 | H19-206 | 0.989 | 1.60 | 7.99 | 0.70 | 3 | LncRNA |
| ENST00000436715 | H19-208 | 0.861 | 1.29 | 4.76 | 0.57 | 5 | Ri |
| ENST00000447298 | H19-211 | 0.592 | 1.32 | 10.63 | 0.90 | 2 | LncRNA |
| ENST00000442037 | H19-210 | 0.798 | 0.80 | 3.49 | 0.64 | 3 | LncRNA |
| ENST00000607600 | AL136131.3-201 | 0.188 | 463.57 | 100.38 | 0.66 | NA | LncRNA |
| ENST00000636484 | RN7SK-201 | 0.328 | 1241.05 | 389.88 | 0.50 | NA | SnRNA |

**(D) Down-regulated noncoding transcripts in urine of high risk patients compared to healthy subjects**

| Transcript ID | Name | length [kb] | HR [TPM] | C [TPM] | log(HR/C) | TSL | Biotype |
|---|---|---|---|---|---|---|---|
| ENST00000625038 | AL008636.1-201 | 1.018 | 387.21 | 596.22 | -0.19 | NA | LncRNA |
| ENST00000521218 | AC103957.2-201 | 0.510 | 588.68 | 740.57 | -0.10 | 2 | LncRNA |
| ENST00000523087 | HDAC2-AS2-217 | 0.493 | 2299.64 | 3.452.42 | -0.18 | 3 | LncRNA |
| ENST00000451415 | HDAC2-AS2-205 | 0.559 | 1982.01 | 3.032.37 | -0.18 | 3 | LncRNA |
| ENST00000387365 | MT-TI-201 | 0.069 | 648.37 | 954.01 | -0.17 | NA | Mt tRNA |
| ENST00000387382 | MT-TW-201 | 0.068 | 1008.28 | 1.383.40 | -0.14 | NA | Mt tRNA |
| ENST00000427161 | AL135790.1-201 | 0.157 | 8300.05 | 11.224.37 | -0.13 | 3 | LncRNA |
| ENST00000562900 | AC009035.1-201 | 0.717 | 1609.17 | 2.115.62 | -0.12 | 3 | LncRNA |
| ENST00000521908 | AC131254.2-201 | 0.550 | 961.94 | 1.247.84 | -0.11 | 3 | LncRNA |
| ENST00000557618 | AL049874.3-201 | 0.868 | 640.98 | 821.81 | -0.11 | 3 | LncRNA |
| ENST00000597502 | AL158835.1-205 | 0.818 | 616.97 | 821.77 | -0.12 | 5 | LncRNA |
| ENST00000611240 | AL158835.1-206 | 0.632 | 528.52 | 676.35 | -0.11 | 5 | LncRNA |
| ENST00000606512 | AC036214.2-201 | 0.917 | 501.33 | 640.63 | -0.11 | NA | LncRNA |
| ENST00000557465 | AL356022.1-201 | 0.483 | 456.93 | 590.92 | -0.11 | 5 | LncRNA |

**(E) Up-regulated circular transcripts in urine of high risk patients compared to healthy subjects**

| Transcript ID | Name | length [kb] | HR [TPM] | C [TPM] | log(HR/C) |
|---|---|---|---|---|---|
| hsa_circ_0037140 | HBA1 | 0.444 | 1090.27 | 66.05 | 1.22 |
| hsa_circ_0037139 | HBA2 | 0.239 | 221.73 | 16.62 | 1.13 |
| hsa_circ_0037141 | HBA1 | 0.239 | 114.55 | 11.04 | 1.02 |
| hsa_circ_0113405 | SLC2A1 | 0.232 | 142.13 | 20.91 | 0.83 |
| hsa_circ_0050104 | GDF15 | 1.200 | 109.04 | 27.23 | 0.60 |
| hsa_circ_0076608 | VEGFA | 1.430 | 303.62 | 77.71 | 0.59 |
| hsa_circ_0014225 | S100A6 | 0.390 | 391.17 | 102.25 | 0.58 |
| hsa_circ_0014226 | S100A6 | 0.683 | 314.28 | 87.06 | 0.56 |
| hsa_circ_0014224 | S100A6 | 0.231 | 105.43 | 29.96 | 0.55 |

**(F) Down-regulated circular transcripts in urine of high risk patients compared to healthy subjects**

| Transcript ID | Name | length [kb] | HR [TPM] | C [TPM] | log(HR/C) |
|---|---|---|---|---|---|
| hsa_circ_0084758 | TRAM1 | 0.176 | 0.66 | 41.33 | -1.80 |
| hsa_circ_0084756 | TRAM1 | 0.383 | 0.81 | 21.56 | -1.43 |
| hsa_circ_0084757 | TRAM1 | 0.447 | 0.98 | 18.51 | -1.28 |
| hsa_circ_0137075 | TRAM1 | 0.380 | 1.15 | 16.15 | -1.15 |
| hsa_circ_0064279 | SEC13 | 0.405 | 14.24 | 140.33 | -0.99 |
| hsa_circ_0065026 | KIF15 | 0.472 | 3.19 | 26.96 | -0.93 |
| hsa_circ_0064275 | SEC13 | 0.591 | 12.43 | 102.34 | -0.92 |
| hsa_circ_0109513 | ZNF382 | 5.901 | 18.56 | 147.16 | -0.90 |
| hsa_circ_0084759 | TRAM1 | 0.422 | 3.08 | 20.04 | -0.81 |

Legend: Protein coding (Pc), Retained Intron (Ri), Processed transcript (Pt), Nonsense mediated decay (NMD), TSL not analyzed (NA), long noncoding RNA (LncRNA), Small nuclear RNA (SnRNA), Mitochondrial transfer RNA (Mt tRNA).

**Supplementary Table 4: Primer pairs and length of PCR amplicons**

| Transcript | 1. Forward Primer 5′→ 3′<br>2. Reverse Primer 5′→ 3′<br>3. TaqMan® probe1 5′→ 3′ | Amplicon length (bp) |
|---|---|---|
| BIRC5 | 1. GACCACCGCATCTCTACATTCA<br>2. CAAGTCTGGCTCGTTCTCAGT<br>3. – | 117 |
| KRT20 | 1. AGCTGCGAAGTCAGATTAAGGA<br>2. GAAGTCCTCAGCAGCCAGTT<br>3. – | 89 |
| LASP1 | 1. CTCGGAACCATGAACCCCA<br>2. ATGCCAGAACTTATCCAGACAGT<br>3. – | 87 |
| OP18 | 1. AAAGACGCAAGTCCCATGAAG<br>2. AGCTTCCATTTTGTGGGTCAG<br>3. 5′-FAM-GCAGCTGGCTGAGAAACGAGAGCA-BHQ1-3′ | 146 |
| UPK1A | 1. CGGAAGGCTGACGTGAAGT<br>2. CGTCATGATTGAGCAAGAATGC<br>3. FAM-TGGCACATCTGGTCCCATGGA-BHQ-1 | 72 |
| 18S rRNA | 1. CACATCCAAGGAAGGCAGCAG<br>2. GACTTGCCCTCCAATGGATCC<br>3. – | 152 |
| CXCL8 | 1. ACCGGAAGGAACCATCTCAC<br>2. GGCAAAACTGCACCTTCACAC<br>3. – | 108 |
| RPS27 | 1. CGAGAACATGCCTCTCGCA<br>2. GCACCAGGCGTTTCTTCTTG<br>3. – | 83 |
| RPL23A | 1. TGCCAACAAAATTGGGATCATCT<br>2. CCCAGCCCAACCAGAAATTG<br>3. – | 115 |
| GLYATL | 1. GAGCATTCGAGAGCACTCCT<br>2. TGGGAGTTTCACTTTCAAATTCATC<br>3. – | 127 |
| ZNF382 | 1. GAGGAGTGGCAGCAACTAGA<br>2. ATGTGAAACCCCACAGATACGA<br>3. – | 95 |
| RYR3 | 1. GTGGGGCTATTCCAGTTGCT<br>2. AAATTGGGTTGGGTCAGGCA<br>3. – | 147 |
| TRAM1 (convergent) | 1. ATCTGGTCAGCTTAGTGCGT<br>2. TGGGGATAAGCCCTCCATAAGA<br>3. – | 111 |
| S100A6 (convergent) | 1. ACTTGGACCGGAACAAGGAC<br>2. AGGGTGTCTCCATCTTCCCT<br>3. – | 126 |
| TRAM1 (divergent) | 1. GTTTGGGGCACATTCATTCTCATC<br>2. GGAGAAGTGCATTCGCCTGT<br>3. – | 113, 320, 363, 384 |
| S100A6 (divergent) | 1. GGGTCCTCTCTGAGTCAAATCCA<br>2. CTGCCGGAGTACTTGTGGAAGA<br>3. – | 150, 443 |

[1]FAM = 6-Carboxyfluorescein; BHQ = Black Hole Quencher™.
